# Hybrid Coatings for Active Protection against Corrosion of Mg and Its Alloys

**DOI:** 10.3390/polym15143035

**Published:** 2023-07-13

**Authors:** Andrey S. Gnedenkov, Sergey L. Sinebryukhov, Valeriia S. Filonina, Alexander Yu. Ustinov, Sergey V. Gnedenkov

**Affiliations:** Institute of Chemistry FEB RAS, Vladivostok 690022, Russia

**Keywords:** magnesium, magnesium alloys, active corrosion protection, corrosion inhibitor, protective coating, plasma electrolytic oxidation (PEO), sodium oleate, polycaprolactone (PCL)

## Abstract

A novel approach to surface modification was developed to improve the corrosion performance of biodegradable magnesium alloys. Additively manufactured magnesium samples and Mg-Mn-based magnesium alloys were used in this study. This method involves the combination of plasma electrolytic oxidation to create a porous ceramic-like matrix, followed by treatment with protective biocompatible agents. The most efficient method for the PEO-layer impregnation using sodium oleate and polycaprolactone was selected and optimized. The correlation between the structure, composition, and protective properties of the hybrid coatings was established. The composition of the formed polymer-containing layers was established using XPS and Raman microspectroscopy. The presence of sodium oleate and its distribution across the coating surface was confirmed at the microscale. The corrosion-protection level of the hybrid layers was assessed using potentiodynamic polarization measurements, electrochemical impedance spectroscopy, hydrogen evolution testing, and gravimetry (mass-loss tests) *in vitro*. The oleate-containing polycaprolactone layers (HC-SO 0.1–2) demonstrated stable corrosion behavior even after 7 days of immersion in Hank’s balanced salt solution. The corrosion-current density and impedance modulus measured at a frequency of 0.1 Hz for the samples with hybrid coating after 7 days of exposure were equal to 5.68 × 10^−8^ A∙cm^−2^ and 2.03 × 10^6^ Ω∙cm^2^, respectively. The developed method of surface modification demonstrates the coating’s self-healing properties. The effectiveness of employing hybrid anticorrosive bioactive PEO coatings for biomedical products made from magnesium and its alloys was demonstrated.

## 1. Introduction

There are numerous requirements for the materials and surface treatments used in biomedicine, particularly for temporary load-bearing devices utilized to fix bone fractures [1,2,3,4]. These requirements include biocompatibility, biodegradability, maintaining surface integrity during the healing process, and stimulating cell adhesion and proliferation [5,6]. Magnesium, as an implant material, fulfills the criteria of both biodegradability and biocompatibility. Its negative standard-electrode potential ensures complete spontaneous degradation in chloride-containing media. Additionally, magnesium is one of the major elements in the human body, meaning it does not exhibit any cellular or tissue toxicity. Moreover, it is reported that the magnesium coronary scaffold Magmaris^TM^, by Biotronik (acquired the CE mark in Europe, approved by FDA in the US and PMDA in Japan [7]) demonstrated greater mechanical properties (e.g., tensile strength and percentage of elongation at break) compared to scaffolds made of poly-L-lactic acid [8]. However, the potential use of magnesium as an implant material faces challenges due to its rapid heterogeneous degradation, resulting in a local pH increase in the surrounding area and the abundant release of hydrogen [9,10]. A high corrosion rate combined with heterogeneous degradation can lead to the breakdown of the implanted Mg-based material. Moreover, the release of H_2_ during the corrosion process significantly reduces the cell adhesion, leading to a slowdown in the healing process and causing implant loosening. The high local alkalization occurring near the implant may provoke an undesirable reaction in the surrounding tissues. To mitigate the corrosion rate of Mg, the formation of protective biocompatible coatings can be employed [4,11,12,13,14,15,16,17,18,19]. For example, clinical studies of drug-loaded DREAMS 2G Biotronik coronary Mg scaffolds coated with PLLA demonstrated an increased resorption time (9 months) compared to items made of commercially pure magnesium (1–3 months [7]) [20]. Nevertheless, in terms of bone fixing, more corrosion-resistant biodegradable materials are required, conditioning the application of other approaches to the surface treatment of magnesium-based products. Plasma electrolytic oxidation (PEO) is a commonly used technology for protection against corrosion in valve metals, including magnesium and its alloys. It facilitates the formation of heterogeneous highly adhesive ceramic-like surface layers with diverse compositions and properties [6,15,18,21,22,23]. Although PEO-layer formation enhances the corrosion resistance of magnesium and its alloys, the heterogeneous morphology of PEO coatings limits its ability to provide the necessary barrier properties throughout the entire healing period (14–17 weeks). The presence of deep pores in the structure of oxide PEO coatings facilitates the penetration of the substrate material by the corrosive solution. This results in the appearance of pitting and the spread of corrosion under the protective layer with further degradation of the substrate. Simultaneously, the pores and microdefects within the PEO coating can be used as microcontainers, retaining and releasing various compounds impregnated in its structure that improve the protective properties of the substrate material. The use of biocompatible and resorbable coating components reduces the corrosive activity of magnesium and its alloys, as well as providing a controlled resorption rate and complete adsorption in the body without harmful effects. Among the compounds suitable for impregnating the porous layers of PEO coatings, many organic corrosion inhibitors not only exhibit biocompatibility but also facilitate the formation of simple surface layers with effective corrosion protection [24]. One such corrosion inhibitor is oleic acid, an omega-9 unsaturated fatty acid present in animal fats and vegetable oils. Its sodium salt, sodium oleate (SO, NaOl), is also recognized as a corrosion inhibitor and demonstrates utility in pharmacology for the development of drug-delivery systems [25,26,27]. Additionally, sodium oleate is also utilized as a corrosion inhibitor for steel, aluminum, and its alloys [28]. For instance, Luo et. al. [29] studied the corrosion behavior of steel in a Na_2_SO_4_ (0.24 mol/L) solution, both before and after treatment with sodium oleate. The findings indicate that the introduction of oleate significantly reduces the electrochemical activity of steel in the studied electrolyte. Chirkunov et. al. [30], formed a PEO coating on an AZ31 magnesium alloy and impregnated it with various inhibitors and their mixtures, including sodium oleate. The inhibitor was inserted into the PEO coating by immersing the samples in a 16-micromolar aqueous solution of sodium oleate for 10 min. The presented results demonstrate that the impregnated samples exhibited a remarkable enhancement in corrosion resistance, as confirmed by electrochemical tests using potentiodynamic polarization and salt-spray-chamber tests.

In our previous work [6], we demonstrated that the combination of the corrosion inhibitor benzotriazole and bioresorbable polymer, both incorporated into the PEO layer, effectively prevents the propagation of corrosion and prevents the further deterioration of the material.

The current work is a continuation of the abovementioned study, focusing on the development of new hybrid coatings containing an inhibitor and a polymer to improve the barrier-anticorrosion properties of bioresorbable magnesium alloys. To provide a prolonged protection and the controlled release of the inhibitor when the protective layer is damaged in a corrosive environment, the microcontainers within the PEO coating were sealed by a biodegradable polymer, polycaprolactone (PCL) [31,32]. Polycaprolactone is an FDA-approved [33,34] biopolymer material, commonly used in surgery and tissue engineering, as well as for the design of drug-delivery systems [35,36,37,38]. Considering the use of the material in medical practice, it can be concluded that PCL provides biocompatibility, an appropriate rate of biodegradation with non-toxic degradation products, processability, and accessibility [31,33,39].

It was observed that there were no previous investigations of the combined effect of sodium oleate and a biodegradable polymer, such as PCL, within the oxide-coating matrix obtained by PEO. This study is intended to establish a method with which to potentially improve the protective properties for magnesium and its alloys, which are promising for implant surgery.

## 2. Materials and Methods

Metal plates with dimensions of 15 × 20 × 1.5 mm, composed of MA8 Mg alloy (weight %: Mn—1.3–2.3, Ce—0.15–0.30, Zn—up to 0.3, Si—up to 0.1, Al—up to 0.1, Fe—up to 0.05, Cu—up to 0.05, Ni—up to 0.007, Be—up to 0.002, Mg—balance) were used for the experiments. The MA8 is a low-alloy Mg-Mn-Ce system that combines high corrosion resistance (compared to commercially pure magnesium), satisfactory mechanical characteristics, good machinability, and affordability [40]. Previous studies showed that MA8 Mg alloy is a promising material for implant surgery [18,41,42,43].

Additionally, investigations were conducted on plates produced through additive technology using direct laser deposition of MPF-4 Mg powder (GOST 6001-79) to form the sample designated hereinafter as AT-Mg. The process used to form AT-Mg samples was described in detail in our previous work [6]. The AT-Mg metal plates with dimensions of 20 × 20 × 0.7 mm were formed in a stainless-steel chamber in Ar (99.993%) atmosphere, ensuring a stable deposition process. Helium was used for Mg-powder supply to the site of deposition. The deposition was performed in 30 passes at a speed of 5 mm/s using a laser beam with a 2.5 mm diameter and a power of 250 W.

The surface preparation of samples involved mechanical grinding using a TwinPrep 5x™ grinder/polisher (Allied High Tech Products, Inc., Compton, CA, USA). Samples were ground using silicone carbide (SiC) papers successively down to P1000 grit (approximately 15 µm abrasive size). The final stage of the specimen preparation included washing with deionized water and drying in the desiccator at 40–45 °C.

The formation of the base oxide coating was accomplished using a PC-controlled plasma electrolytic oxidation (PEO) unit. The specimens were treated in aqueous solution of 25 g/L Na_2_SiO_3_ and 7 g/L NaF, employing a combined bipolar mode in two steps [6]. The first step included a potential increase in the anode component in the range of 30–280 V, as well as potential stabilization of the cathodic component at −40 V. First-stage duration was 200 s, sweep rate was 1.4 V/s. In the second stage, the potential of the anode component was reduced to 200 V (0.2 V/s) vs. previous step. The cathode component was also changed potentiodynamically to −10 V (0.075 V/s). Total oxidation time was 600 s and the duty cycle was 50%.

During the course of the presented study, composite coatings (CC) were formed on the base of the porous PEO-layer, which included either sodium oleate or polycaprolactone, as well as hybrid coatings (HC) that incorporated both the corrosion inhibitor and polymer. Treatment of the porous part of base PEO layer with sodium oleate was conducted using aqueous solutions with inhibitor concentration of 0.05 M and 0.1 M. pH of the solutions was neutralized (7.0–7.3) by acidification with 0.1 M HCl. Impregnation of the PEO coating was performed by immersing the specimens in the solutions, followed by vacuum treatment by means of Epovac vacuum-impregnation equipment (5 min) (Struers A/S, Copenhagen, Denmark) to eliminate air from the pores. Subsequently, the specimens were immersed in the solutions for 1 h in air under constant stirring to enhance the deposition of the inhibitor within the porous part of a PEO layer. After impregnation, the inhibitor-containing samples were gradually extracted from the solution, dried in air, and treated with the polycaprolactone (6 wt.% in dichloromethane solution) to seal the microcontainers and prevent premature release of the inhibitor from the pores.

The polymer layer was formed by immersing the inhibitor-loaded PEO sample in the polymer-containing solution for 5–7 s and then slowly withdrawing it. The polymer application was performed twice. As an alternative to the layer-by-layer treatment, hybrid coatings were formed by impregnation of the PEO-treated sample with solution of sodium oleate (concentrations were 0.05 M and 0.1 M) and 6 vol.% polycaprolactone in dichloromethane. The impregnation of the heterooxide matrix with the resulting solution was also carried out by means of vacuum-impregnation apparatus. All modified samples were subsequently dried at 40–45 °C for 24 h [6].

Since the presented work is devoted to the design of novel coating types using the application of different modifying agents, it is necessary to estimate the level of protective properties of different systems, containing only a corrosion inhibitor and a polymer material, or their mixture in the PEO-coating matrix. Various samples were used during the study in order to select the optimal concentrations of inhibitor and polymer, as well as to find the best method of combination of these protective agents. These specimens were used for a comparative evaluation of the level of the anticorrosion protection of the formed coatings and assessment of the efficacy of the proposed method of surface treatment of magnesium alloys. The samples prepared for the experiments are designated in Table 1.

The morphology of the obtained sample surface and elemental composition were studied by means of scanning-electron microscopy (SEM), along with energy-dispersive spectroscopy (EDX). Merlin Gemini II electron microscope (Carl Zeiss, Jena, Germany) equipped with X-MaxN Silicon Drift Detector Sdd 80 (Oxford Instruments NanoAnalysis, Concord, MA, USA) was used. Metallographic cross-sections of the studied samples were prepared for SEM-EDX studies.

The sample cross-sections were prepared by cold-pouring the specimens with epoxy resin and subsequently processed using a Tegramin-25 grinder/polisher (Struers A/S, Copenhagen, Denmark). The grinding and polishing stages involved the use of abrasive papers, polishing discs [44], and diamond suspensions with grain sizes of 9–3 µm, along with DP-Lubricant Brown and OP-S NonDry colloidal suspension.

The phase composition of the formed PEO-layers was determined by X-ray-diffraction analysis (XRD). SmartLab diffractometer (Rigaku, Tokyo, Japan) with CuK_β_ radiation was used. The spectra were recorded at a room temperature within the range of 2θ = 4°–90°, with a step of 0.01°. The generator current and voltage were 140 mA and 42 kV, respectively.

The chemical composition of CC-SO 0.1 sample was analyzed using X-ray photoelectron spectroscopy (XPS). The surface was examined at 0.5 µPa using non-monochromatic AlKα radiation (1486.6 eV). The measurements were conducted using the SPECS (Germany) spectrometric complex, employing a hemispherical energy analyzer PHOIBOS-150. Calibration of all spectra was performed versus the carbon (C1s) with a binding energy equal to 285.0 eV. The studied area underwent etching with an Ar^+^ beam for 600 s (5000 eV) in scanning mode, and a layer approximately 5–10 nm thick was removed. This step was used to investigate the chemical composition of the subsurface layers of the anticorrosion coating and clean the area under study.

The impregnation of the PEO-coating structure with sodium oleate (CC-SO 0.1) was confirmed using confocal Raman microspectroscopy. The spectra were obtained using a green laser (532 nm) with an acquisition time of 600 s (60 accumulated spectra). The measurements were conducted in the range of 300 to 3300 cm^−1^ with a power output of 35 mW. The Alpha500 confocal spectrometer (WITec, Ulm, Germany) was used. Raman spectra were analyzed using WITec Control software. The selected area of the coating with a size of 35 × 30 μm was scanned, resulting in 35 × 35 spectra. During scanning, the integration time for recording spectra was equal to 1 s.

The corrosion properties were evaluated using potentiodynamic polarization (PDP) test and electrochemical impedance spectroscopy (EIS), along with monitoring of open circuit potential (OCP) in the 0.9% NaCl solution. This medium was used for corrosion tests of Mg samples that were promising for implant surgery, since 0.9% NaCl solution is a physiological solution with a chloride concentration equal to Cl^−^ contents in the blood. The experiments were conducted by means of VersaSTAT MC system (PAR, San Jose, CA, USA). The scanned surface area was 1 cm^2^. The platinized niobium mesh was applied as a counter electrode. The reference electrode was the silver chloride (Ag/AgCl) electrode with a potential of 0.197 V (vs. the NHE). Before the PDP and EIS tests, the specimen was kept in the solution for 1 h to achieve a steady state. The sweep rate during PDP testing was 1 mV/s, and anodic polarization of the sample was carried out in the range of potentials from −0.25 V up to +0.5 V versus *E*_C_ (where *E*_C_ is the corrosion potential). The impedance spectra were recorded in the frequency range of 1 MHz–0.1 Hz, employing a logarithmic sweep with 10 data points per decade. Moreover, the impedance spectra were recorded during the 23 h of exposition of the sample to electrolyte. This duration was suitable for a preliminary express estimation of the corrosion behavior of studied samples.

To simulate the corrosion behavior of specimens with protective coatings in environments that mimicked human-body medium, long-term electrochemical tests were conducted in Hank’s balanced salt solution (HBSS) for 7 days. The EIS spectra were obtained every 2 h during the first 48 h and every 4 h thereafter. After the last EIS measurement (after 23 h of immersion in 0.9 wt.% NaCl solution and 7 days of exposure to HBSS), the PDP test was also performed.

The Levenberg–Marquardt approach, known for its suitability for describing electrochemical parameters of valve metals like Mg and its alloys, was applied to determine the values of the corrosion potential (*E*_C_) and corrosion-current density (*I*_C_) by means of Butler–Volmer Equation (1). [45,46]. Values of the polarization resistance (*R*_P_) were measured in accordance with [47].

(1) $$I=I_{c}\left(10^{\frac{E-E_{c}}{\beta_{a}}}+10^{-\frac{E-E_{c}}{\beta_{c}}}\right)$$

The inhibitor efficiency was estimated in accordance with Equation (2).

*η*_i_ = ((*I*_c0_ − *I*_c_)/*I*_c0_) × 100%, (2)

where *I*_c0_ and *I*_c_ are the values of the corrosion-current density measured for a system without and with inhibitor, respectively.

To determine the corrosion-degradation rate of specimens with protective coatings, gravimetric (mass-loss tests) and volumetric (hydrogen evolution tests) methods were employed. Using these techniques, the material’s weight loss and the volume of evolved hydrogen per unit area of the specimen were measured during immersion in Hanks’ solution for 7 days.

The mass loss of the material due to corrosion was estimated using the gravimetric method. Corrosion products formed during the immersion were removed by washing the samples in deionized water for 15 min using the ultrasonic bath. The washed samples were weighed using AUW120D analytical balance (Shimadzu, Kyoto, Japan).

For hydrogen-evolution tests, an eudiometer (2591-10-500, Neubert-Glas, Geschwenda, Geratal, Germany) was used, ensuring the isolation of the test solution from the air.

Four samples with a total surface area of 28 cm^2^ were used simultaneously for volumetric/gravimetric tests. The experiments were conducted at room temperature and under constant stirring in the 500 mL solution [48]. All experiments were conducted in triplicates to ensure reliability of the data. The relative error in the volume of evolved H_2_ did not exceed 10%. After the exposure period, the specimens were removed from the solution, washed with deionized water, and subsequently dried in air. Based on the results of hydrogen-evolution tests, the corrosion rate (*P*$V_{H_{2}}$) of the studied samples was estimated according to the equation presented in [49].

In order to investigate the diffusion process of the inhibitor’s release from the composite (CC-SO 0.1) and hybrid coatings (HC-SO 0.1–2), high-performance liquid chromatography (HPLC) was employed. Five specimens of each type (with dimensions of 1.5 × 2.0 × 0.15 cm) were immersed in 300 mL of a 0.9 wt.% NaCl solution for a 21 days. Every day, 3 mL of the solution was collected to evaluate the concentration of the inhibitor released during the sample immersion. The HPLC analysis was conducted using a Shimadzu LC-20A liquid chromatograph equipped with a UV detector SPD-20A (detector wavelengths were set to 210 nm and 275 nm) and a low-temperature laser-light scattering detector ELSD-LT II. The component separation was performed using Agilent Eclipse XDB-C18 column (4.6 × 150 mm, 5 μm). The temperature of the column was maintained at 40 °C. A mobile phase consisting of a 50:50 mixture of methanol and water was used. The flow of the mobile phase was set at a rate of 16 μL/s.

## 3. Results

**Figure 1:**
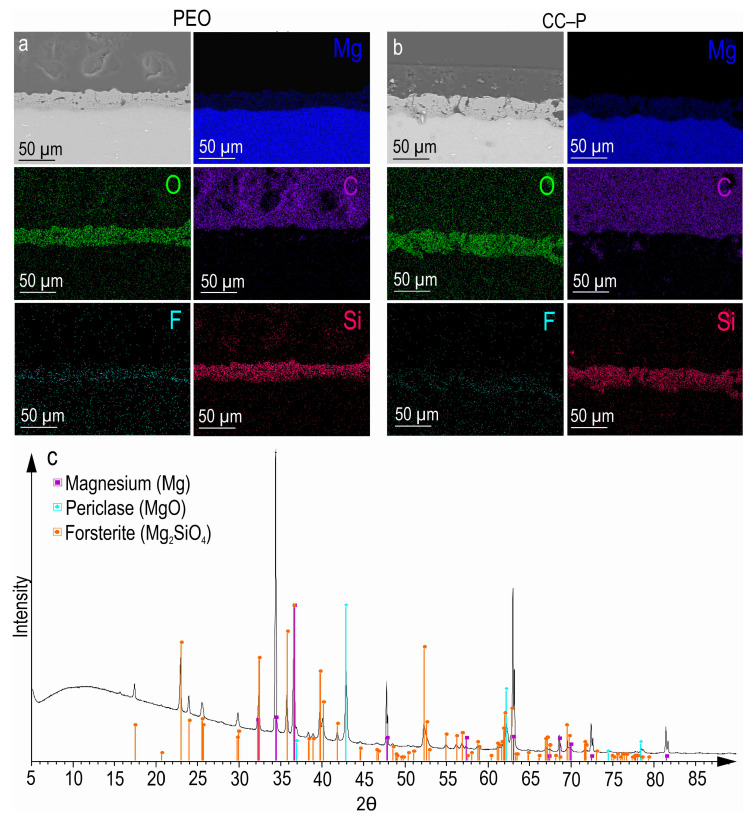
SEM images and maps of EDX analysis of element distribution within the cross-sections of PEO (**a**) and CC-P (**b**) samples and XRD pattern of the PEO sample (**c**).

**Figure 2:**
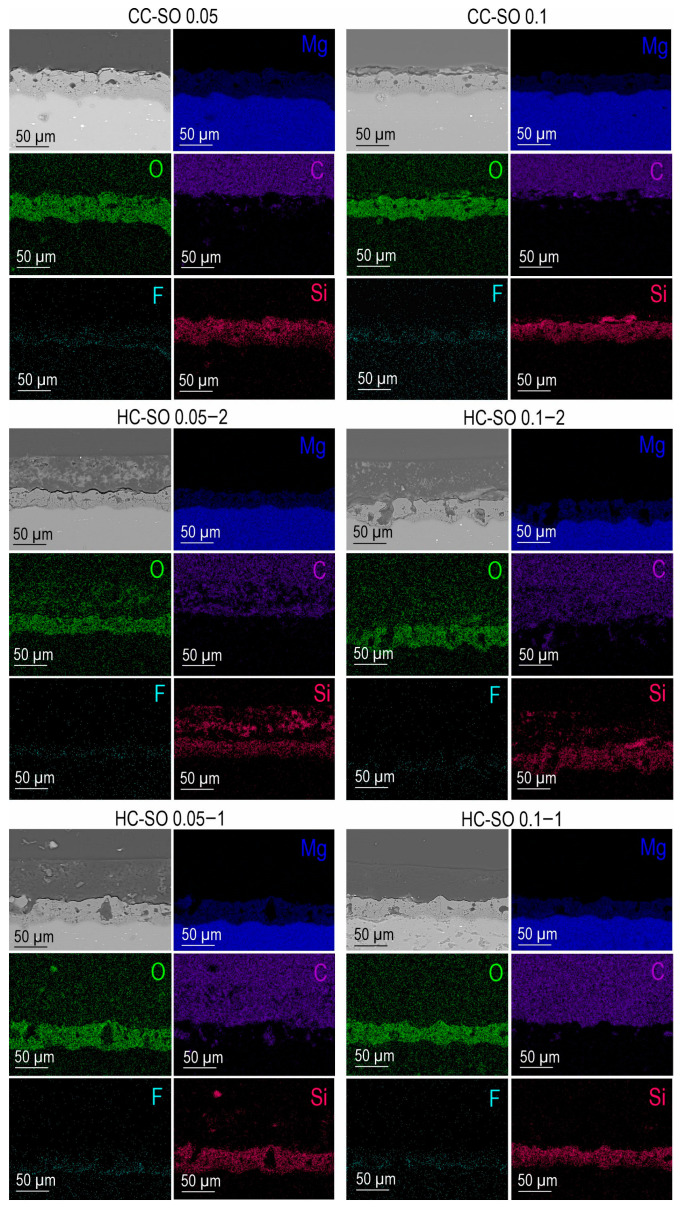
SEM images and EDX maps of element distribution within the cross-sections of MA8 alloy samples with various coatings.

After the PEO treatment, a porous ceramic-like coating was formed on the surface of the MA8 magnesium alloy. The composition of the resulting oxide layer, as indicated by the SEM-EDX and XRD analyses (Figure 1a,c), revealed the presence of Mg, O, F, and Si elements in the compounds, including MgO and Mg_2_SiO_4_ (Figure 1c). An examination of SEM images and EDX maps showing the distribution of the elements within the coating’s thickness indicated that the pores of the PEO coating were filled with corrosion-inhibitor and polymer components (Figure 1b and Figure 2). It can be inferred that the impregnation of the PEO layer with PCL effectively reduced the likelihood of the penetration of aggressive corrosive substances into the substrate.

**Figure 3:**
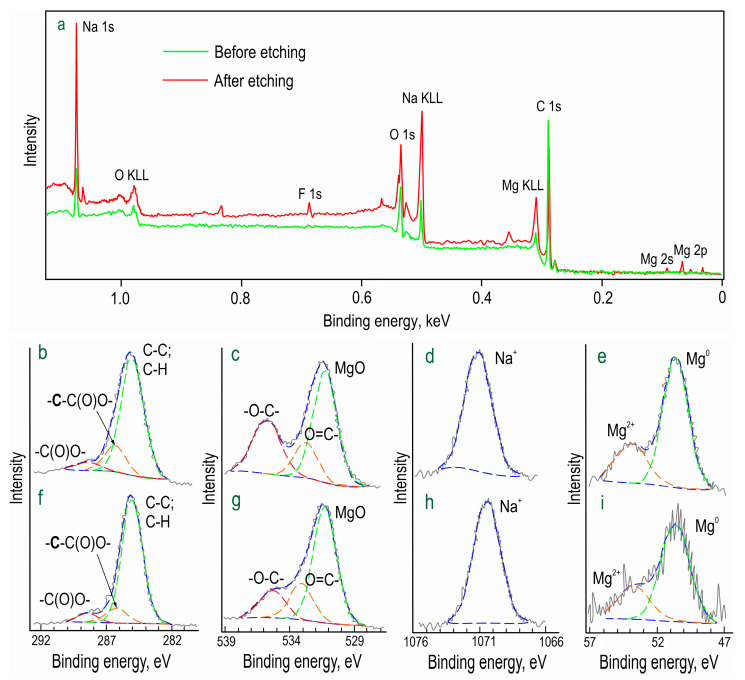
XPS spectra of the CC-SO 0.1 sample (**a**). The C 1s (**b**,**f**), O 1s (**c**,**g**), Na 1s (**d**,**h**), and Mg 2p (**e**,**i**) high-resolution spectra before (**b**–**e**) and after (**f**–**i**) Ar^+^ etching.

The chemical composition of formed inhibitor-containing protective layer (CC-SO 0.1) was studied by means of X-ray photoelectron spectroscopy (XPS) (Figure 3; Table 2). It was revealed that, in addition to a high concentration of aliphatic carbon, this element, with a binding energy of 288 eV, was in the oxidized state (-C(O)O-). Moreover, the carbon with the binding energy of 286 eV belonged to the methylene group (-CH_2_-), which is consistent with the structure of sodium oleate. The spectra indicated that both the upper layer (prior to the etching) and the underlying layer contained substantial amounts of oxygen and sodium. This composition is attributable to the presence of the corrosion inhibitor in the PEO layer. Oxygen with binding energies of 535 and 533 eV indicated the presence of -O-C- and O=C- bonds, respectively, in the sodium oleate.

The relatively low magnesium content suggested the uniform distribution of the inhibitor over the PEO coating. The abundance of sodium, however, indicated the presence of forms distinct from sodium oleate. The increased sodium content after the etching can be attributed to the composition of the PEO coating. Taking into account that all oxygen at 535 eV and ca. 4 at. % of sodium is associated with sodium oleate, the results of the XPS analysis confirmed that C_63_H_115.5_O_7_Na_3.5_ was one of the main components of the upper layer in the CC-SO 0.1 sample. This substance had a composition closely matching that of sodium oleate stoichiometry.

**Figure 4:**
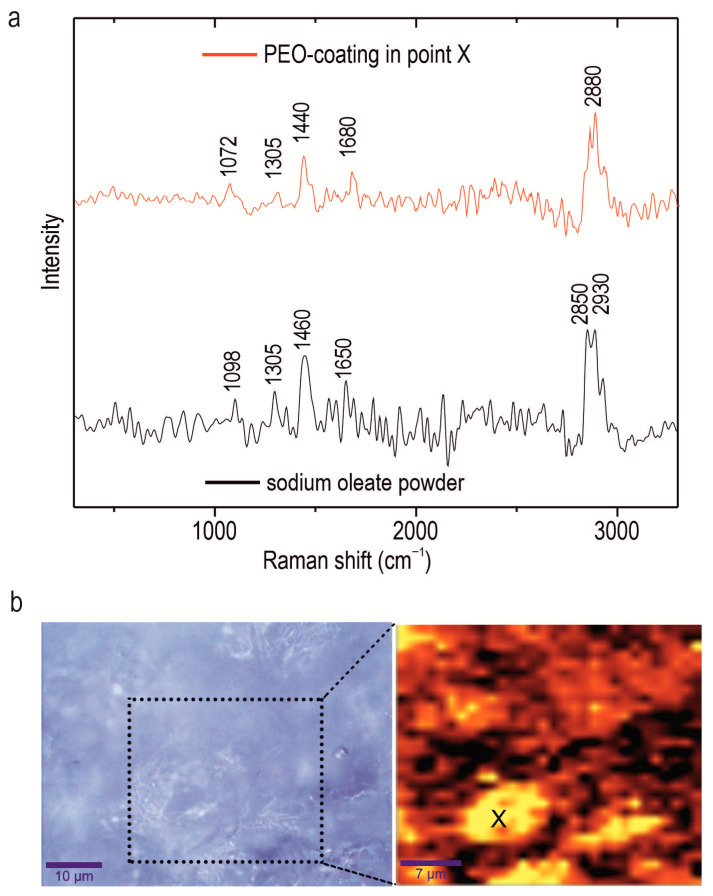
Raman spectrum of sodium oleate powder and spectrum from the area marked with X in (**b**) of CC-SO 0.1 (**a**), optical image (on the left side of (**b**)), and 2D inhibitor-distribution map (on the right side of (**b**)).

Confocal Raman microspectroscopy was employed to investigate the chemical composition of an alloy with a composite oleate-containing coating, the CC-SO 0.1 sample. This method enables the determination of components’ distribution within the protective layer at the microscale in scanning mode. The Raman spectrum of the sodium oleate powder is depicted in Figure 4. Notably, a broad band within the range of 2840–2940 cm^−1^ was observed, indicating the stretching vibrations of the ν(C-H) bond. Specifically, asymmetric stretching vibrations (-CH_2_-) were detected at 2850 cm^−1^, while symmetric stretching vibrations ν(-CH_3_-) were observed at 2930 cm^−1^ [50,51]. The peak at 1098 cm^−1^ represents the bending vibrations of the C-C bond within the hydrocarbon chain of the sodium oleate [50]. The peaks at 1305 cm^−1^ and 1460 cm^−1^ are attributable to the aliphatic chain of the sodium oleate, indicating bending vibrations of δ(CH_2_) [52,53]. The peak at 1650 cm^−1^ is related to the stretching vibration of ν(C=O) bond [53]. Figure 4b represents an optical image of the analyzed area (enclosed within a frame), along with the corresponding intensity distribution of the sodium oleate in the CC-SO 0.1 sample. To create a 2D inhibitor-distribution map, the Raman-shift range of 2770–2960 cm^−1^, encompassing the stretching vibrations of the ν(C-H) bond, was used. The analysis of the experimental data revealed a high concentration of sodium oleate on the sample surface. The intensity map illustrates areas with a uniform distribution of sodium oleate, as well as agglomerates containing the substance, which appear as lighter regions. The presence of a corrosion inhibitor on the surface of the PEO coating served as an additional barrier layer, thereby enhancing the sample’s anticorrosive properties. The surface area where the Raman spectrum (shown in Figure 4a) was acquired is marked by the “X” symbol in Figure 4b. The Raman spectrum displays distinctive bands at 1072, 1305, 1440, 1680, and 2880 cm^−1^, corresponding to the sodium oleate. These findings indicate heterogeneity in the inhibitor distribution over the sample surface.

**Figure 5:**
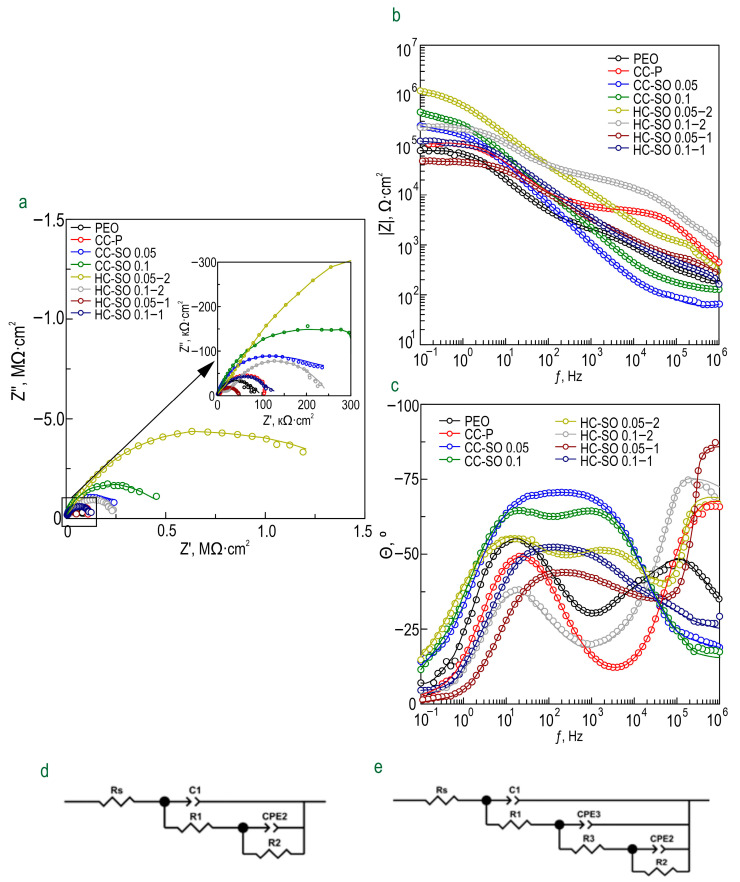
Impedance spectra (Nyquist (**a**) and Bode (**b**,**c**) plots), obtained after 1 h immersion of samples with studied coatings in 0.9% NaCl. Equivalent electrical circuits (EECs) used to fit the EIS spectra (**d**,**e**).

**Figure 6:**
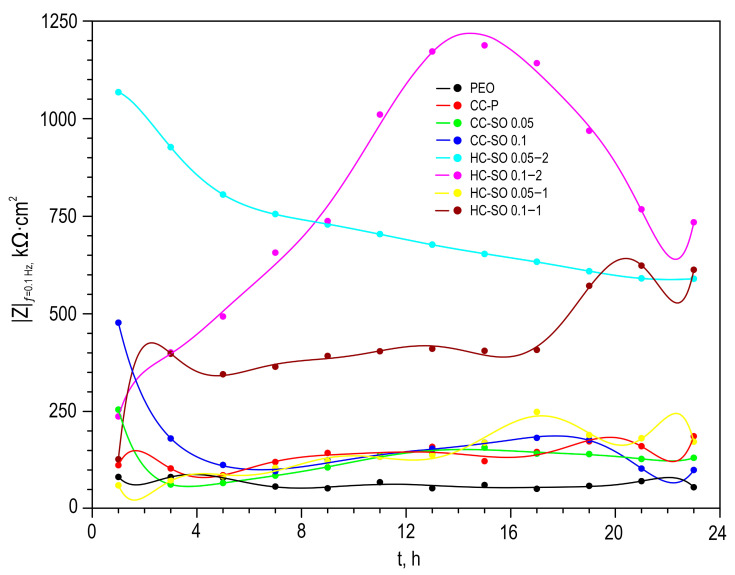
The change in the impedance modulus detected at *f* = 0.1 Hz versus time of the exposure of investigated specimens to 0.9 wt.% NaCl solution.

**Figure 7:**
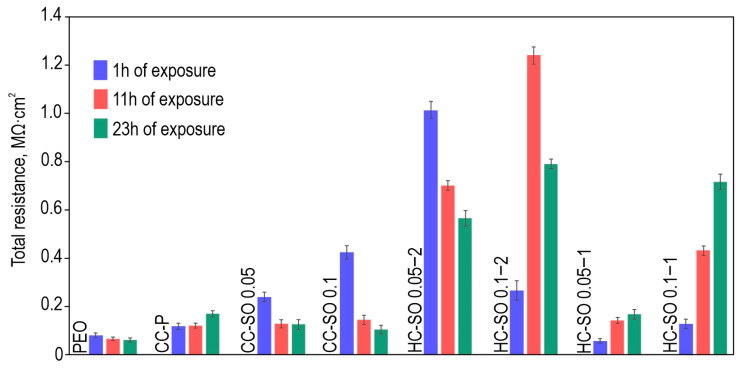
Evolution of the calculated total resistance of the samples with formed surface layers during the immersion in 0.9 wt.% NaCl solution.

The evolution of the protective properties of the specimens with the PEO layer, the composite polymer-containing (CC-P) and inhibitor-containing (CC-SO 0.05, CC-SO 0.1) coatings, and the samples with hybrid coatings (HC-SO 0.05/0.1–2, HC-SO 0.05/0.1–1) during the long-term exposure (23 h) to the corrosive environment was studied by EIS. Figure 5 displays the impedance spectra, presented as Nyquist and Bode plots. Additionally, Figure 6 presents the evolution of the |*Z*|_*f* = 0.1 Hz_ (impedance modulus measured at a low frequency, *f* = 0.1 Hz) during the specimen’s immersion in 0.9% NaCl solution. The phase angle vs. frequency plots for the PEO, CC-P, CC-SO 0.05, CC-SO 0.1, HC-SO 0.1–2, and HC-SO 0.05–1 samples exhibited two time constants (Figure 5c). Therefore, the impedance spectra were fitted using equivalent electrical circuits (EEC) comprising two series-parallel *R-CPE* chains (Figure 5d). To account for the heterogeneity of the studied protective layers, the constant phase element (*CPE*) was utilized as a representation of the ideal capacitance in the analysis. The *R*_1_*–CPE*_1_ chain represents the resistance (*R*_1_) of the porous layer of the formed PEO coating impregnated with sodium oleate at various concentrations (CC-SO 0.05, CC-SO 0.1) and the polymer component (CC-P, HC-SO 0.05–2, HC-SO 0.1–2) or an inhibitor–polymer solution (HC-SO 0.05–1), as well as the geometrical capacitance (*CPE*_1_) of the whole coating (applicable to all types of coating). The *R*_2_*–CPE*_2_ chain describes the resistive and capacitive behavior of the inner sublayer (the non-porous barrier part of the coating), considering the presence of the inhibitor and the polymer deposited in the PEO coating pores. Furthermore, for the impedance spectra obtained during the exposure of the HC-SO 0.1–1 sample, the *θ-ƒ* diagrams demonstrate the presence of three time constants (Figure 5c). Consequently, the fitting of the impedance spectra was conducted by means of EEC with *R-CPE* chains (depicted in Figure 5e). Taking into consideration the limited solubility of sodium oleate in dichloromethane, the hybrid coatings, HC-SO 0.05–1 and HC-SO 0.1–1, exhibited undissolved NaOl powder particles on their surfaces. It is likely that these particles dissolve in the electrolyte, leading to changes in the morphology of the surface layer. Additionally, the corrosion inhibitor dissolved in the electrolyte further enhanced the protective properties of the specimen during exposure to the NaCl solution. This was achieved through a reduction in the electrochemical reactions rate due to the subsequent interaction with the material’s surface and deposition at the pitting area. Moreover, the swelling of the polymer during exposure to the corrosive environment may have contributed to changes in the morphology of the upper layer. Partial degradation, flaking, and the sealing of the porous part of the coating may have occurred. The combination of these factors resulted in the appearance of the third time constant for the HC-SO 0.1–1 sample, unlike the HC-SO 0.05–1 specimen. In the latter case, the effect of the dissolution of the corrosion inhibitor precipitated at the coating’s surface was negligible due to its lower concentration, which stimulated better solubility in dichloromethane and the formation of a more uniform initial surface layer. Consequently, for the HC-SO 0.1–1, the *R*_1_–*CPE*_1_ chain describes the resistance of the upper layer (including the electrolyte resistance in the pores) formed by the inhibitor and polymer deposited on the surface and within the porous part of the PEO layer, and also reflects the geometric capacitance of the whole coating. The *R*_2_–*CPE*_2_ chain represents the inner non-porous barrier layer of the formed coating, comprising the inhibitor–polymer composition deposited at the bottom of the pores. Finally, the *R*_3_–*CPE*_3_ chain describes the inner porous part of the composite coating covered with a polymer/inhibitor. For all the hybrid oleate-containing coatings, slight changes were observed in the values of *Q* (*CPE* coefficient) and *n* (an exponential factor reflecting the approximation of element properties to conductivity (*n*→0) or capacitance (*n*→1)) during the sample exposure (Table S1). These changes indicate slight variations in the coating configuration, including morphology, composition, and properties). The resistance values of the inner (*R*_2_, *R*_3_) and outer (*R*_1_) sublayers of the coating showed changes during exposure to the aggressive destructive environment, suggesting the manifestation of self-healing properties by the coatings. A comparative analysis of the corrosion-resistance results of the formed surface layers revealed the positive effect of incorporating the inhibitor into the coating composition on its protective properties. The impregnation of the pores in the PEO coating with sodium oleate at concentrations of 0.05 M (CC-SO 0.05; |*Z*|_*f* = 0.1 Hz_ = 242,810 Ω‧cm^2^) and 0.1 M (CC-SO 0.1; |*Z*|_*f* = 0.1 Hz_ = 457,030 Ω‧cm^2^) led to an increase in the impedance modulus measured at a frequency *f* = 0.1 Hz by 3.2 and 5.9 times, respectively, compared to the values obtained for the inhibitor-free PEO layer (|*Z*|_*f* = 0.1 Hz_ = 76,910 Ω‧cm^2^). However, the direct contact between the modifying agent and the electrolyte stimulated the premature release of the inhibitor from the oxide PEO layer, resulting in decreased barrier properties in the inhibitor-containing coating. This may have led to an early reduction in the corrosion resistance of the implant and, potentially, to the partial loss of its mechanical integrity. These processes may negatively affect osteosynthesis by provoking the formation of undesirable mechanical stresses in the surrounding bone tissue. Therefore, it is necessary to seal nano- and microreservoirs containing the inhibitor with a bioresorbable polymeric material, such as polycaprolactone. The treatment of the CC-SO 0.05 sample with polycaprolactone (HC-SO 0.05–2) resulted in a significant increase in the level of corrosion protection (Figure 5, Table S1). The value of |*Z*|_*f* = 0.1 Hz_ for these surface layers reached 1,024,800 Ω‧cm^2^, which was 9.7 times higher than that obtained for the CC-P (|*Z*|_*f* = 0.1 Hz_ = 105,880 Ω‧cm^2^). Among the oleate-containing coatings, the HC-SO 0.1–2 sample demonstrated the highest resistance to the corrosion processes (Figure 5 and Figure 6, Table S1). The maximum recorded |*Z*|_*f* = 0.1 Hz_ value for this type of coating was 1,140,200 Ω‧cm^2^ (after 15 h of immersion in the NaCl solution), which was more than nine times higher than the |*Z*|_*f* = 0.1 Hz_ value at the corresponding exposure stage in the corrosive environment for the CC-P sample (|*Z*|_*f* = 0.1 Hz_ equal to 115,870 Ω‧cm^2^). Additionally, this type of coating exhibited the highest polarization resistance after long-term exposure to the aggressive environment (*R*_p_ = 2.99 × 10^7^ Ω‧cm^2^, Table 3). Figure 7 illustrates the changes in the calculated total resistance (*R*_1_ + *R*_2_ + *R*_3_) for the investigated samples, obtained by fitting the impedance spectra after 23 h of immersion in the NaCl solution using an appropriate EEC. It can be inferred that the protective properties of the PEO sample decreased over time due to the partial degradation of the porous coating. The slight increase in total resistance in the CC-P coating can be attributed to the polymer component swelling in the formed surface layer. However, the anticorrosion properties of the polymer-containing composite coating were still insufficient to provide long-term protection against corrosion. The samples with composite-inhibitor-containing coatings (CC-SO 0.05, CC-SO 0.1) exhibited higher levels of corrosion protection than the PEO and CC-P during the initial hour of the experiment. This result can be attributed to the early manifestation of active corrosion-protection properties upon direct contact between the coated sample and the aggressive medium. The HC-SO 0.05–2 sample as characterized by strong protective properties at an early stage of its immersion in the electrolyte (1 h, Figure 5, Figure 6 and Figure 7). This can be explained by the premature release of the corrosion inhibitor from the formed microcontainers and the manifestation of self-healing properties. However, during the analysis of the evolution of the total resistance and impedance modulus measured at a low frequency during the 23 h of exposure to the 0.9 wt.% NaCl solution, it was revealed that the HC-SO 0.05–2 sample was characterized by a significant decrease in corrosion resistance, which indicated insufficient inhibitor concentration in the studied system. The low inhibitor concentration in the pores of the PEO layer can also be related to its partial release into the solution during the formation of the polymer layer. In turn, the HC-SO 0.1–2 was characterized by the most noticeable expression of self-healing properties upon long-term contact with the aggressive environment. The data presented for the HC-SO 0.1–2 sample suggest that this method of forming hybrid coatings best fulfills the objectives of the research. At the initial stage of the test (after 1 h of immersion), partial degradation of the PCL layer was observed, followed by a gradual release of the inhibitor from the pores (11 h of exposure), resulting in a significant increase in the level of anticorrosion protection (Figure 7). The results obtained for the hybrid coatings formed by the one-step treatment of the PEO sample in the inhibitor and polymer solution in dichloromethane (HC-SO 0.05–1, HC-SO 0.1–1) indicate lower inhibitor efficiency compared to the coatings formed via a two-step process. This result shows that PEO-coating impregnation with an inhibitor followed by polymer treatment provides higher anticorrosion protection than the formation of an inhibitor-containing PCL-layer on the surfaces of PEO-treated alloys.

The changes in the protective properties during the immersion of the HC-SO 0.1–2 sample in the sodium chloride solution (Figure 6 and Figure 7) indicate the most pronounced manifestation of self-healing properties among all the studied coatings containing NaOl. According to [54], the periosteum initially forms and stabilizes a bone defect 7–10 days after bone injury during the bone-formation phase, prior to the final phase of bone remodeling. Therefore, the long-term effect of the corrosion protection of formed hybrid coating contributes to the maintenance of the implant’s mechanical strength, minimizing mechanical stresses at the interface between the Mg material and the bone, and optimizing tissue formation [55,56].

The corrosion parameters of the formed surface layers obtained using the potentiodynamic polarization test (Figure S1) and electrochemical impedance spectroscopy (Figure 5, Figure 6 and Figure 7) are presented in Table 3. Based on a comparative analysis of the obtained data, it can also be concluded that the hybrid inhibitor-containing coatings demonstrated the highest corrosion resistance of all the studied coatings. These coatings exhibited the lowest corrosion-current density and the highest impedance modulus at a frequency of 0.1 Hz after 1 h and 23 h of exposure. The samples with hybrid inhibitor- and polymer-containing coatings (HC-SO 0.1–2) were also characterized by the highest inhibitor efficiency, which reached 99.4% after 23 h of immersion in the 0.9% NaCl solution (Table 3).

**Figure 8:**
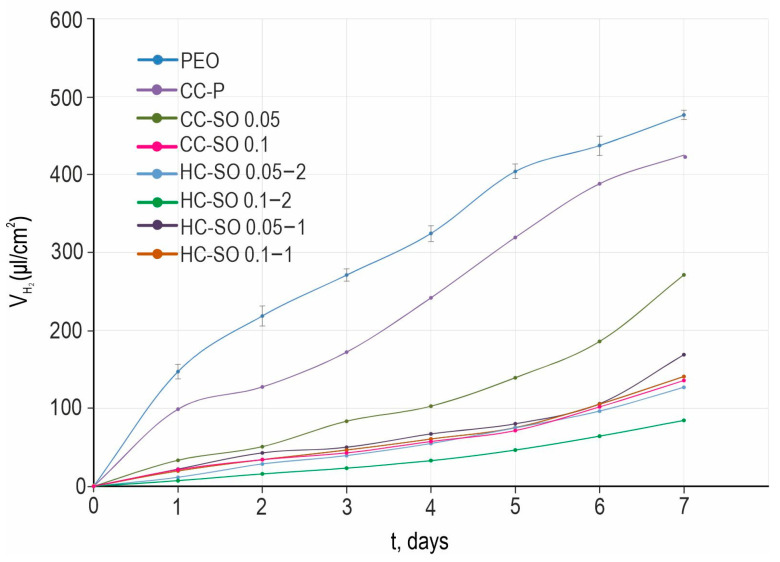
Change in the volume of released hydrogen ($V_{H_{2}}$) normalized to the area of the sample surface for MA8 Mg alloy with different coatings during 7 days of immersion in HBSS medium.

The analysis of the results of the H_2_-evolution tests (Figure 8) revealed an improvement in the protective properties of the formed composite and hybrid coatings with inhibitors in comparison with the PEO and CC-P specimens. The PEO sample exhibited the highest amount of released hydrogen ($V_{H_{2}}$) per surface-area unit (480 μL/cm^2^). However, the PEO-coating impregnation with sodium oleate and polycaprolactone resulted in a significant decrease in the amount of released hydrogen volume during the exposure to HBSS (Figure 8). This outcome highlights the positive impact of surface treatments with corrosion inhibitors and polymer materials. A comparison of the volumetry and EIS data (with exposure to the 0.9 wt.% NaCl solution and HBSS) indicated their consistency. Of the anticorrosion coatings, the hybrid coating formed by the PEO-coated sample’s impregnation with the 0.1 M NaOl and polycaprolactone solution in two stages exhibited the best protective properties. The total volume, $V_{H_{2}}$, was equal to 85 μL/cm^2^ for the HC-SO 0.1–2 sample, which was 6.6 times lower than that obtained for the PEO sample. Despite the variations in the hydrogen-evolution rate during the exposure period, the $V_{H_{2}}$ values were similar among all the samples with inhibitor-containing protective coatings. This demonstrates the low rate of degradation of the materials with anticorrosion coating. According to the data presented in Table 4, the treatment of a PEO-coated sample (*P*$V_{H_{2}}$ = 0.14 mm/y) with 0.05 M and 0.1 M solutions of NaOl leads to a decrease in corrosion rates by 1.5 (*P*$V_{H_{2}}$ = 0.09 mm/y) and 3.7 times (*P*$V_{H_{2}}$ = 0.038 mm/y), respectively. The lowest corrosion rate after 7 days of the immersion of the studied samples in the HBSS was that of the HC-SO 0.1–2. The *P*$V_{H_{2}}$ of the samples with this type of coating was equal to 0.025 mm/y, which was 5.6 times lower than that of the PEO-coated specimens (*P*$V_{H_{2}}$ = 0.14 mm/y).

Based on the mass-loss tests, the corrosion-degradation rate of the specimens (mg cm^−2^ day^−1^) was calculated, considering the exposure time in the Hanks solution (Table S2). A comparative analysis of the experimental data revealed that the samples with hybrid inhibitor-containing coatings exhibited high corrosion resistance during the long-term (7-day) immersion in the corrosive environment. Specifically, samples with HC-SO 0.1–2 demonstrated the lowest rate of corrosion degradation (0.0132 ± 0.0026 mg cm^−2^ day^−1^), which was 3.1 times lower than the corrosion rate for the sample with the PEO coating (0.0415 ± 0.0042 mg cm^−2^ day^−1^). These findings are consistent with the results obtained through the PDP and EIS tests, utilizing exposure to the 0.9 wt.% NaCl solution.

In a sample with a composite coating containing sodium oleate, the inhibitor release was observed only on the 14th day of exposure. The maximum concentration of sodium oleate in the solution was below 0.00005 M. However, during the immersion of the sample with the hybrid coating, the inhibitor release could not be confirmed by HPLC. This is attributable to the low concentration of the inhibitor in the solution, which fell below the detection limit for this substance. The obtained results indicate the effective protection provided by the formed coating containing the inhibitor and polymer.

**Figure 9:**
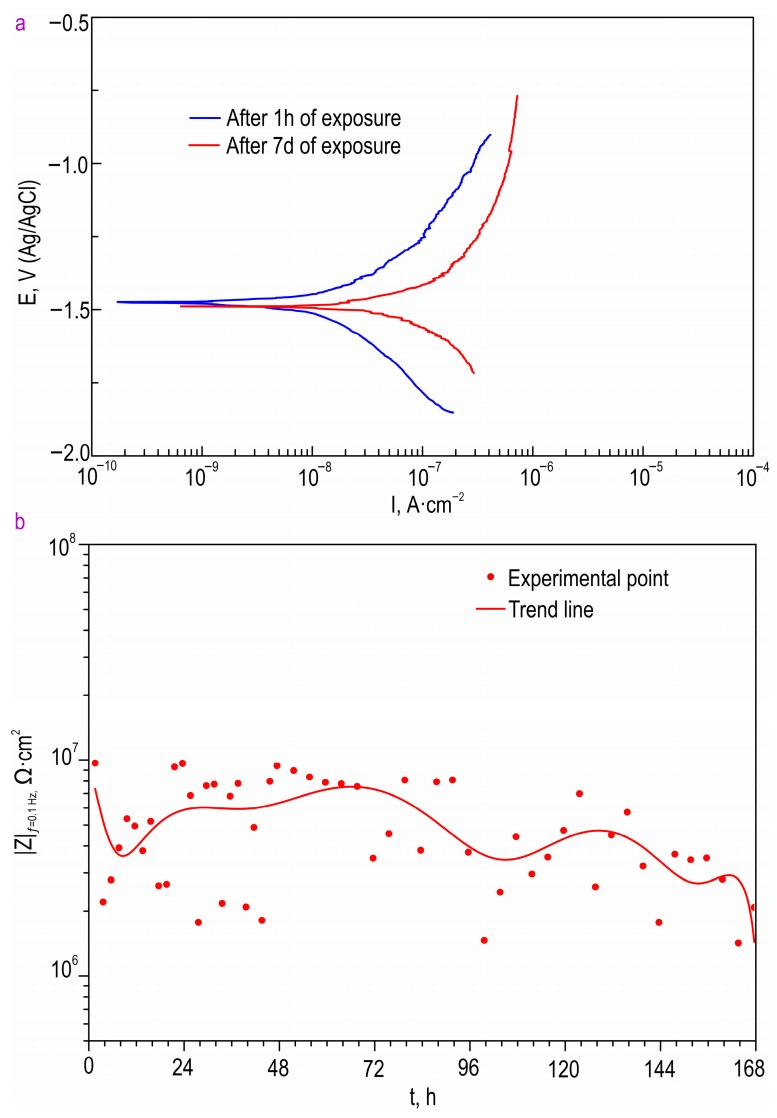
Polarization curves (**a**) and the change in the impedance modulus, detected at the frequency of 0.1 Hz, during the immersion of HC-SO 0.1–2 samples in Hanks’ solution (**b**).

The PDP data obtained from the long-term exposure of the HC-SO 0.1–2 sample in Hanks’ balanced salt solution are presented in Figure 9 and Table 5. Based on these data, it is evident that the sample with the hybrid coating containing the inhibitor and polymer, HC-SO 0.1–2, exhibited a low corrosion rate in the physiological solution. The corrosion-current density for the HC-SO 0.1–2 sample was measured as 1.29 × 10^−8^ A∙cm^−2^. The changes in the impedance modulus, detected at the lowest frequency, |*Z*|_*f* = 0.1 Hz_, over the exposure period of the samples to Hanks’ solution (Figure 9) clearly demonstrate the evolution of the barrier properties of the coatings during prolonged contact with a medium simulating human blood plasma. Through a comparative analysis of the collected data, it was established that the samples with the hybrid coating exhibited consistent electrochemical behavior in the tested medium. According to the presented data, the oleate-containing hybrid coatings demonstrated the highest corrosion resistance during the initial 5 days of exposure. Subsequently, there was a slight decrease in the level of anticorrosion protection, followed by stabilization. These findings are consistent with the calculated EEC parameters obtained by fitting the impedance spectra recorded during the 7-day exposure to Hanks’ solution (Table 6). The parameters *CPE* and *R* showed insignificant changes, further confirming the high resistance to corrosion of the specimens with hybrid layers. From the experiment, it can be inferred that hybrid coatings are capable of providing the necessary level of corrosion protection, ensuring the mechanical integrity of implanted materials during the initial phase of bone formation [54].

The evaluation of the PDP-study results following the exposure of the samples to Hanks’ solution for 7 days confirmed the effectiveness of the incorporation the inhibitor into the composition of the protective coatings (Table 5). The corrosion-current density for the HC-SO 0.1–2 sample slightly increased after 7 days of exposure (5.68 × 10^−8^ A∙cm^−2^), compared to the corresponding value obtained after 1 h of exposure to the harsh environment. This indicates the presence of self-healing properties in the formed surface layers. Such hybrid coatings ensure stable, prolonged, and active protection for MA8 Mg alloys against destructive external influences.

The oleate-containing surface layer exhibited excellent barrier properties, reducing the penetration of the aggressive environment. Through the partial degradation of the upper polymer layer, the adsorbed inhibitor effectively lowered the degradation rate of the material, leading to the slower formation of the corrosion-product layer. This effect contributed to the enhancement of the corrosion resistance of the material compared to systems in which the formation of surface films occurs more intensively.

**Figure 10:**
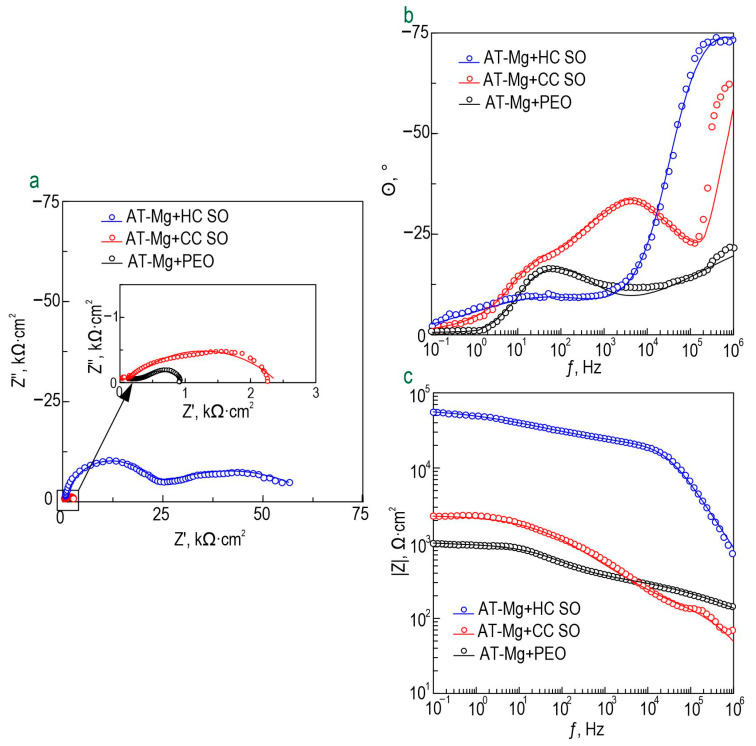
Impedance spectra (Nyquist (**a**) and Bode (**b**,**c**) plots), obtained after 1 h exposure to 0.9% NaCl solution of AT-Mg samples with different types of formed coating.

**Figure 11:**
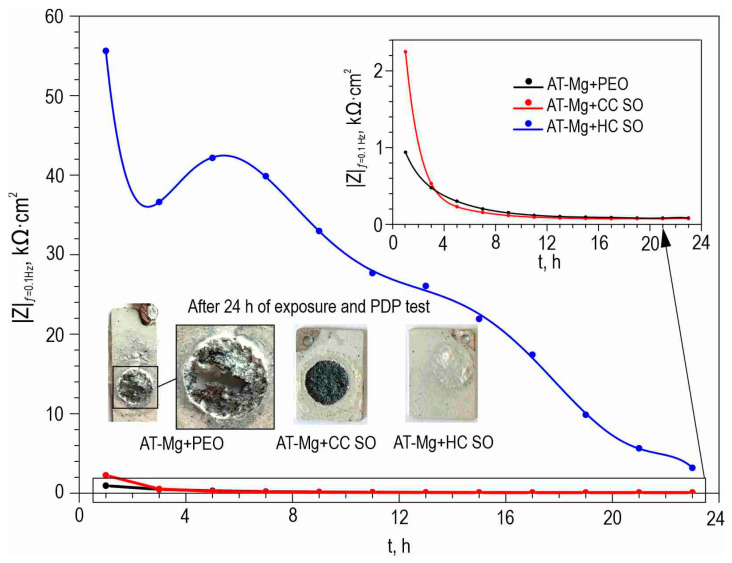
The change in the impedance modulus detected at the lowest frequency (|*Z*|_*f* = 0.1 Hz_) versus the exposure time of AT-Mg with various types of coating to the 0.9% NaCl solution. Optical images of sample surfaces after 23 h of immersion in a sodium chloride solution are also provided.

**Figure 12:**
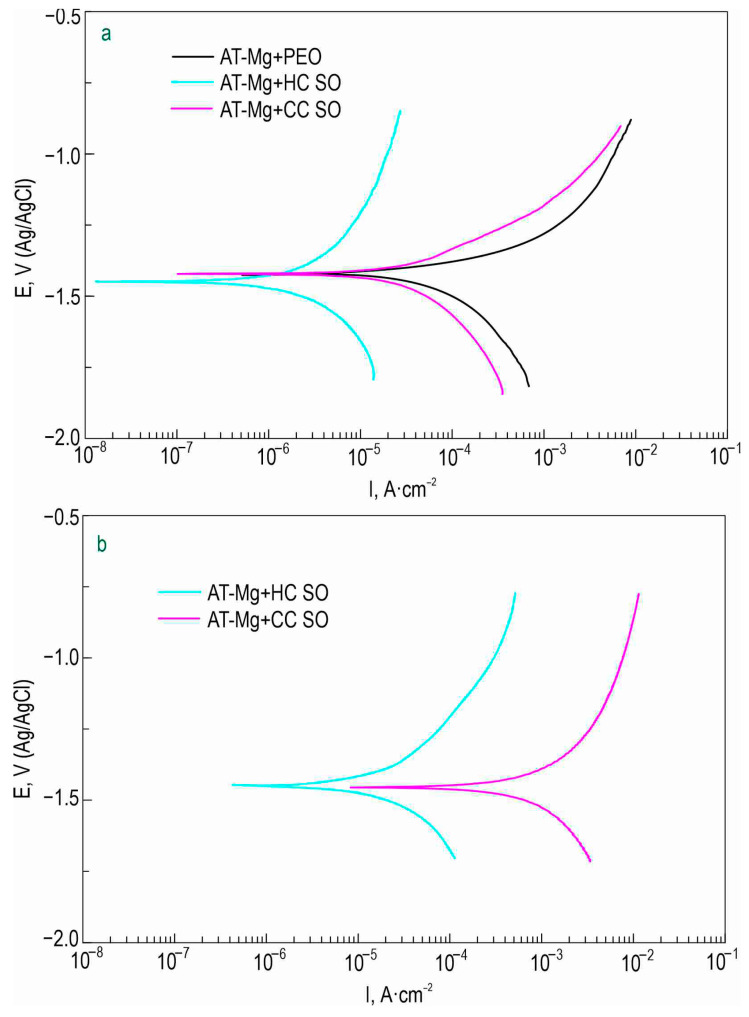
PDP curves obtained for AT-Mg specimens with various types of coating after 1 h (**a**) and 23 h (**b**) of immersion in 0.9 wt.% solution of sodium chloride.

The anticorrosion properties of the coatings obtained on the surface of the Mg sample produced by direct laser deposition were determined through the analysis of the impedance spectra presented in Figure 10. The Bode plots for all the studied samples exhibited two distinct time constants, which necessitated the use of the equivalent electrical circuits with two series-parallel *R-CPE* chains for fitting the experimental impedance spectra (Figure 5d). The parameters (*R_N_*) and (*CPE_N_*) represented the characteristics of the protective layers formed on the Mg. The changes in the calculated parameters of the EEC elements and the impedance modulus detected at *f* = 0.1 Hz for the AT-Mg+PEO and oleate-containing samples during the 23 h of exposure to the 0.9 wt. % NaCl solution are presented in Table S3 and Figure 11, respectively. The impedance value measured at the lowest frequency (|*Z*|_*f* = 0.1 Hz_) for the AT-Mg+CC SO sample exhibited an increase of more than twofold (|*Z*|_*f* = 0.1 Hz_ = 2249 Ω·cm^2^) compared to the AT-Mg+PEO specimen (|*Z*|_*f* = 0.1 Hz_ = 940 Ω·cm^2^). A comparative analysis of the calculated data obtained from the potentiodynamic polarization test (Figure 12, Table 7) also revealed the positive influence of the sodium oleate on the corrosion resistance of the AT-Mg. The corrosion-current density of the AT-Mg+CC SO sample (*I*_C_ = 3.91 × 10^−5^ A·cm^−2^) was 2.9 times lower than that of the PEO-coated specimen (*I*_C_ = 1.14 × 10^−4^ A·cm^−2^). The anticorrosion properties of the AT-Mg+CC SO sample were maintained even after 23 h of exposure to the chloride-containing medium (*I*_C_ = 3.82 × 10^−4^ A·cm^−2^), in contrast to the AT-Mg+PEO specimen, which corroded during exposure to the NaCl (Figure 11). The structure of the metal substrate was not uniform, nor was that of the heterooxide layer that formed on its surface. The PEO treatment of the AT-magnesium reduced the specific surface area of the material in contact with the aggressive medium. However, the heterogeneity of the microstructure of the substrate material and the complex morphology of the formed protective coating resulted in the formation of numerous initiation centers for corrosion processes. It is worth noting that the calculation of the EEC parameters for the specimens with AT-Mg+PEO and AT-Mg+CC SO after 5 h of immersion was not possible as a result of the significant decrease in the corrosion resistance and the poor quality of the obtained spectra. However, the AT-Mg+HC SO sample showed no violation of its mechanical integrity after the long-term exposure and polarization (Figure 11). These results indicate the positive effect of modifying the base PEO coating by means of a corrosion inhibitor. The AT-Mg+HC SO demonstrated the highest resistance to the corrosion processes. The values of |*Z*|_*f* = 0.1 Hz_ for this coating was more than 60 times higher (|*Z*|_*f* = 0.1 Hz_ = 55,630 Ω·cm^−2^) and the *I*_C_ for the hybrid coating (*I*_C_ = 3.71 × 10^−8^ A·cm^−2^) was more than three orders of magnitude lower, than the equivalent parameters for the sample with the base PEO layer (|*Z*|_*f* = 0.1 Hz_ = 940 Ω·cm^2^, *I*_C_ = 1.14 × 10^−4^ A·cm^−2^) (Figure 10, Table 7). The AT-Mg+CC SO sample also exhibited a slight change in its *Q* and *n* parameters during the exposure time, which indicated slight changes in the morphology, composition, and properties of the coating. It was observed that the impregnation of the PEO-coated AT-Mg with the corrosion inhibitor and polymer solution significantly enhanced the resistance of the inner (*R*_1_) and outer (*R*_2_) layers of the protective coating. The decrease in these parameters during the sample’s exposure to the corrosive environment is attributable to the partial degradation of the protective coating. Nevertheless, the hybrid coating demonstrated superior electrochemical resistance, especially after immersion in a 0.9% sodium chloride solution for 23 h. The corrosion-current density, *I*_C_, for the AT-Mg+HC SO sample after the exposure was equal to 2.25 × 10^−5^ A·cm^−2^, which was more than an order of magnitude lower than the value of this parameter for the AT-Mg+CC SO (*I*_C_ = 3.82 × 10^−4^ A·cm^−2^).

Therefore, according to the analysis of the experimental data from the EIS, PDP, hydrogen evolution, and mass-loss tests, the HC-SO 0.1–2 (for MA8 alloy) and AT-Mg+HC SO (for AT-Mg) samples exhibited the strongest anticorrosive properties. These hybrid coatings formed via the sequential impregnation of the PEO-coated samples in solutions with NaOl and PCL provided the MA8 magnesium alloy and the AT-Mg with the highest levels of corrosion protection. The use of this surface treatment makes it possible to implement the controlled resorption of Mg-based implants while maintaining their mechanical integrity throughout the entire healing process.

## 4. Conclusions

Studies were conducted with the aim of developing a method to modify the surface of the bioresorbable magnesium alloy, MA8, and magnesium samples obtained with additive technology (AT-Mg). The objective was to create hybrid coatings that incorporate an organic corrosion inhibitor and a biodegradable polymer material, with the goal of reducing corrosion degradation. The following findings were obtained from the study.

The plasma-electrolytic oxidation of the MA8 and AT-Mg surfaces resulted in the formation of ceramic-like coatings with well-developed surfaces. The X-ray-diffraction analysis revealed the presence of forsterite (Mg_2_SiO_4_) and periclase (MgO) in the composition of the coating. The surface relief provides a suitable matrix for incorporating modifying agents, such as biocompatible corrosion inhibitors and polymers.

The method was selected and optimized to achieve the most effective impregnation of the pores in the PEO coating with the corrosion inhibitor and polymer. Hybrid coatings were formed via the sequential impregnation of the PEO-coated samples in solutions with various concentrations of NaOl and PCL (HC-SO 0.05–2, HC-SO 0.1–2, AT-Mg+HC SO), as well as via the one-stage application of PCL and NaOl, in a dichloromethane solution (HC-SO 0.05–1, HC-SO 0.1–1).

The presence of sodium oleate in the composition of the composite-inhibitor-containing coatings (CC-SO 0.05, CC-SO 0.1) was confirmed using X-ray photoelectron spectroscopy, and its distribution within the coatings was established by means of confocal Raman microspectroscopy.

The level of corrosion protection provided by the coatings on the MA8 Mg alloy and AT-Mg was assessed. The HC-SO 0.1–2 exhibited the strongest anticorrosive properties, according to the EIS and PDP data. The maximum recorded |*Z*|_*f* = 0.1 Hz_ value for this specimen was 1,140,200 Ω‧cm^2^ (after 15 h of exposure to NaCl solution). This sample also exhibited the highest polarization resistance (*R*_p_ = 2.99 × 10^7^ Ω‧cm^2^) and the highest inhibitor efficiency (99.4%) after long-term exposure to the aggressive environment.

The electrochemical behavior of the HC-SO 0.1–2 sample with the hybrid coating was evaluated during its exposure to HBSS. The corrosion-current density and impedance modulus detected at a low frequency for the samples with hybrid coatings after 7 days of exposure were equal to 5.68 × 10^−8^ A∙cm^−2^ and 2.03 × 10^6^ Ω∙cm^2^, respectively. This indicates the effectiveness of the developed method of surface modification and the manifestation of self-healing properties.

The volumetric and gravimetric tests revealed that the HC-SO 0.1–2 sample exhibited the lowest volume of released hydrogen ($V_{H_{2}}$ = 85 μL/cm^2^) and the lowest weight loss (0.0132 ± 0.0026 mg‧cm^−2^‧day^−1^) of all the studied layers. These samples also demonstrated the lowest corrosion rate (0.025 mm/y) after 7 days of immersion in HBSS.

The HPLC data indicated that the impregnation of the pores of the PEO layer using a polymeric material significantly reduced the amount of inhibitor released into the solution. This confirmed the efficacy of the proposed surface-modification method for bioresorbable magnesium and its alloys.

The optimal approach to hybrid-coating formation via the sequential impregnation of PEO-coated samples was established. The role of each component of these smart coatings (e.g., corrosion inhibitor, polymer) in enhancing the corrosion resistance was highlighted. It was revealed that the combination of sodium oleate and polycaprolocatone improves the corrosion behavior of PEO-treated Mg and its alloys. This study established the effectiveness and potential of using hybrid coatings containing a bioresorbable polymer material and a biocompatible corrosion inhibitor for the controlled resorption of biomedical products made of magnesium and its alloys.

The future research directions based on this study include the monitoring of long-term electrochemical stability in various specific environments (e.g., Hanks’ balanced salt solution and minimum essential medium) to improve the accuracy of the prediction of the corrosion behavior of samples with the studied types of protective layer *in vitro*, as well as the performance of *in vivo* studies to confirm the biocompatibility of the formed material.

## Figures and Tables

**Table 1:** Designation of the samples with coatings formed in the presented work and their descriptions.

Designation	Description
PEO	MA8 Mg alloy specimen with the base PEO coating
CC-P	MA8 specimen with the composite coating formed by treating the PEO sample in the solution of the 6 vol.% polycaprolactone in dichloromethane
CC-SO 0.05	MA8 specimen with the composite coating formed by treating the PEO sample in the aqueous solution of sodium oleate (0.05 M)
CC-SO 0.1	MA8 specimen with the composite coating formed by treating the PEO sample in the aqueous solution of sodium oleate (0.1 M)
HC-SO 0.05–2	MA8 specimen with the hybrid coating formed by treating the PEO sample in the aqueous solution of sodium oleate (0.05 M), followed by immersion in the solution of 6 vol.% polycaprolactone in dichloromethane
HC-SO 0.1–2	MA8 specimen with the hybrid coating formed by treating the PEO sample in the aqueous solution of sodium oleate (0.1 M), followed by immersion in the solution of 6 vol.% polycaprolactone in dichloromethane
HC-SO 0.05–1	MA8 specimen with the hybrid coating formed by treating the PEO sample in the solution of sodium oleate (0.05 M) and polycaprolactone (6 vol.%) in dichloromethane
HC-SO 0.1–1	MA8 specimen with the hybrid coating formed by treating the PEO sample in the solution of sodium oleate (0.1 M) and polycaprolactone (6 vol.%) in dichloromethane
AT-Mg+PEO	AT-Mg specimen with the base PEO coating
AT-Mg+CC SO	AT-Mg specimen with the composite coating formed by treating the PEO sample in the aqueous solution of sodium oleate (0.1 M)
AT-Mg+HC SO	AT-Mg specimen with the hybrid coating formed by treating the PEO sample in the aqueous solution of sodium oleate (0.1 M), followed by immersion in the solution of 6 vol.% polycaprolactone in dichloromethane

**Table 2:** The binding energy (in eV) and elemental composition (in at %, enclosed in brackets) of the PEO-coated sample treated with sodium oleate (CC-SO 0.1).

Studied Surface	Na (1s)	O (1s)			C (1s)			Mg (2p)	
Before etching	1070.4 (6.0)	535.3 (2.7)	533.0 (3.1)	531.3 (8.7)	288.4 (5.7)	286.2 (8.3)	285.0 (63.9)	53.3 (0.4)	50.7 (1.2)
After etching	1071.3 (16.5)	535.9 (7.0)	532.6 (3.8)	531.2 (9.6)	288.4 (4.9)	286.2 (10.7)	285.0 (45.4)	53.9 (0.5)	50.6 (1.6)
Chemical state	Na^+^	-O-C-	SiO_2_ O=C-	MgO	-C(O)O-	-C-C(O)O-	C-C, C-H	Mg^2+^	Mg^0^

**Table 3:** The electrochemical parameters obtained from the analysis of PDP curves and EIS spectra, and inhibitor efficiency after 1 h and 23 h of sample immersion in a 0.9 wt.% NaCl solution.

Sample	*β*_a_, mV/Decade	−*β*_c_, mV/Decade	*I*_C_, A·cm^−2^	*E*_C_, V (Ag/AgCl)	*R*_P_, Ω·cm^2^	|*Z*|*_f_*_= 0.1 Hz_, Ω·cm^2^	*η*_i_ *, %*
PEO after 1 h of exposure	220.70	218.18	2.40 × 10^−6^	−1.50	1.99 × 10^4^	76,910	– *
PEO after 23 h of exposure	Coating breakdown					60,522	– *
CC-P after 1 h of exposure	268.36	270.80	7.55 × 10^−7^	−1.46	7.76 × 10^4^	105,880	– *
CC-P after 23 h of exposure	346.87	237.49	4.63 × 10^−7^	−1.46	1.32 × 10^5^	177,130	– *
CC-SO 0.05 after 1 h of exposure	238.73	141.02	3.45 × 10^−7^	−1.33	1.12 × 10^5^	242,810	85.6
CC-SO 0.05 after 23 h of exposure	286.61	143.80	7.34 × 10^−8^	−1.37	5.67 × 10^5^	124,060	– *
CC-SO 0.1 after 1 h of exposure	105.31	205.36	5.39 × 10^−8^	−1.32	1.55 × 10^6^	457,030	97.8
CC-SO 0.1 after 23 h of exposure	159.10	93.61	4.26 × 10^−8^	−1.38	6.02 × 10^5^	93,942	– *
HC-SO 0.05–2 after 1 h of exposure	379.11	160.13	6.11 × 10^−8^	−1.44	8.01 × 10^5^	1,024,800	91.0
HC-SO 0.05–2 after 23 h of exposure	139.35	76.63	1.99 × 10^−8^	−1.47	1.08 × 10^6^	565,150	95.7
HC-SO 0.1–2 after 1 h of exposure	181.39	115.64	1.71 × 10^−8^	−1.28	1.80 × 10^6^	225,960	97.7
HC-SO 0.1–2 after 23 h of exposure	447.19	378.21	2.98 × 10^−9^	−1.07	2.99 × 10^7^	764,370	99.4
HC-SO 0.05–1 after 1 h of exposure	151.41	171.59	2.23 × 10^−7^	−1.51	1.57 × 10^5^	56,227	70.4
HC-SO 0.05–1 after 23 h of exposure	141.31	99.046	6.43 × 10^−9^	−1.29	3.94 × 10^6^	163,770	98.6
HC-SO 0.1–1 after 1 h of exposure	108.59	105.48	1.38 × 10^−8^	−1.34	1.68 × 10^6^	124,470	98.2
HC-SO 0.1–1 after 23 h of exposure	103.36	101.91	6.23 × 10^−9^	−1.29	3.58 × 10^6^	620,100	98.7

* The inhibitor efficiency that cannot be calculated.

**Table 4:** Corrosion rate of the studied samples after 7 days of exposure to HBSS.

Sample	PEO	CC-P	CC-SO 0.05	CC-SO 0.1	HC-SO 0.05–2	HC-SO 0.1–2	HC-SO 0.05–1	HC-SO 0.1–1
*P*$V_{H_{2}}$, mm/y	0.14	0.13	0.09	0.038	0.036	0.025	0.05	0.04

**Table 5:** Calculated corrosion performance for HC-SO 0.1–2 samples after 1 h and 7 days of exposure to HBSS.

Sample Type	*β*_a_, mV/Decade	−*β*_c_, mV/Decade	*I*_C_, A·cm^−2^	*E*_C_, V (Ag/AgCl)	*R*_P_, Ω·cm^2^	|*Z*|_*f* = 0.1 Hz_, Ω·cm^2^
After 1 h exposure	258.36	307.41	1.29 × 10^−8^	−1.49	4.72 × 10^6^	9.39 × 10^6^
After 7 d exposure	276.28	245.43	5.68 × 10^−8^	−1.50	9.94 × 10^5^	2.03 × 10^6^

**Table 6:** The EEC parameters obtained by fitting the EIS spectra of HC-SO 0.1–2 samples during the exposure to HBSS for 7 days.

Exposure Time, h	*CPE*_1_		*R*_1_, Ω·cm^2^	*CPE*_2_		*R*_2_, Ω·cm^2^
	*Q*_1_, S·cm^−2^·s^n^	*n*_1_		*Q*_2_, S·cm^−2^·s^n^	*n*_2_	
1	1.41 × 10^−10^	0.99	1.83 × 10^6^	2.36 × 10^−9^	0.50	6.99 × 10^6^
3	1.57 × 10^−10^	0.97	1.26 × 10^6^	3.72 × 10^−7^	0.47	1.33 × 10^6^
11	1.63 × 10^−10^	0.97	1.98 × 10^6^	6.75 × 10^−8^	0.69	3.50 × 10^6^
23	2.88 × 10^−10^	0.96	4.64 × 10^6^	8.84 × 10^−8^	0.43	4.32 × 10^6^
47	4.17 × 10^−10^	0.92	5.75 × 10^6^	1.92 × 10^−7^	0.46	5.21 × 10^6^
71	4.03 × 10^−10^	0.92	2.68 × 10^6^	1.30 × 10^−6^	0.59	2.33 × 10^6^
119	3.83 × 10^−10^	0.92	2.32 × 10^6^	2.56 × 10^−7^	0.58	3.80 × 10^6^
167	2.50 × 10^−10^	0.95	1.26 × 10^6^	1.89 × 10^−6^	0.30	1.34 × 10^6^

**Table 7:** The electrochemical parameters obtained from the PDP and EIS tests for coated AT-Mg samples after 1 h and 23 h of immersion in a 0.9% NaCl solution.

Sample Type	*β*_a_, mV/Decade	−*β*_c_, mV/Decade	*I*_C_, A·cm^−2^	*E*_C_, V (Ag/AgCl)	*R*_P_, Ω·cm^2^	|*Z*|_*f* = 0.1 Hz_, Ω·cm^2^
AT-Mg+PEO after 1 h exposure	275.45	556.01	1.14 × 10^−4^	−1.45	7.02 × 10^2^	940
AT-Mg+PEO after 23 h exposure	– *	– *	– *	– *	– *	82
AT-Mg+CC SO after 1 h of exposure	154.94	279.75	3.91 × 10^−5^	−1.42	1.11 × 10^3^	2249
AT-Mg+CC SO after 23 h of exposure	156.68	174.19	3.82 × 10^−4^	−1.45	9.40 × 10^1^	75
AT-Mg+HC SO after 1 h of exposure	465.28	213.07	3.71 × 10^−8^	−1.35	1.71 × 10^6^	55,630
AT-Mg+HC SO after 23 h of exposure	299.47	259.20	2.25 × 10^−5^	−1.45	2.69 × 10^3^	3182

* The electrochemical parameters that cannot be accurately calculated.

## Data Availability

Not applicable.

## Supplementary Materials

The following supporting information can be downloaded at: https://www.mdpi.com/article/10.3390/polym15143035/s1, Figure S1: Polarization curves obtained after 1 h (a) and 24 h (b) of exposure of the studied samples to 0.9% NaCl solution; Table S1: The calculated parameters of the EEC elements, obtained by fitting the experimental impedance spectra of the coated samples during their exposure to a 0.9% NaCl solution; Table S2: The results of gravimetric measurements of specimens with different types of coatings after 7 days immersion in HBSS; Table S3: The calculated parameters of the EEC elements, obtained by fitting the experimental impedance spectra of the coated AT-Mg samples during their exposure to 0.9% NaCl solution for 24 h.
