# Supplementary material for: Hybrid Coatings for Active Protection against Corrosion of Mg and Its Alloys
Source: Polymers (Basel). 2023 Jul 13;15(14):3035. doi: 10.3390/polym15143035 (PMC10384155; doi:10.3390/polym15143035)
Supplement: Supplementary file 1 [file polymers-15-03035-s001.zip › polymers-2477655-supplementary.pdf]

polymers
MDPI

Supplementary Materials

Hybrid Coatings for Active Protection against Corrosion of Mg and Its Alloys

Andrey S. Gnedenkov *, Sergey L. Sinebryukhov, Valeriia S. Filonina, Alexander Yu. Ustinov and Sergey V. Gnedenkov

Institute of Chemistry FEB RAS, Vladivostok, 690022, Russia; sls@ich.dvo.ru (S.L.S.); filonina.vs@gmail.com (V.S.F.); all_vl@mail.ru (A.Y.U.); svg21@hotmail.com (S.V.G.)
* Correspondence: asg17@mail.com; Tel.: +8-(423)-2215284; Fax: +8-(423)-2312590

SUPPLEMENTARY MATERIALS

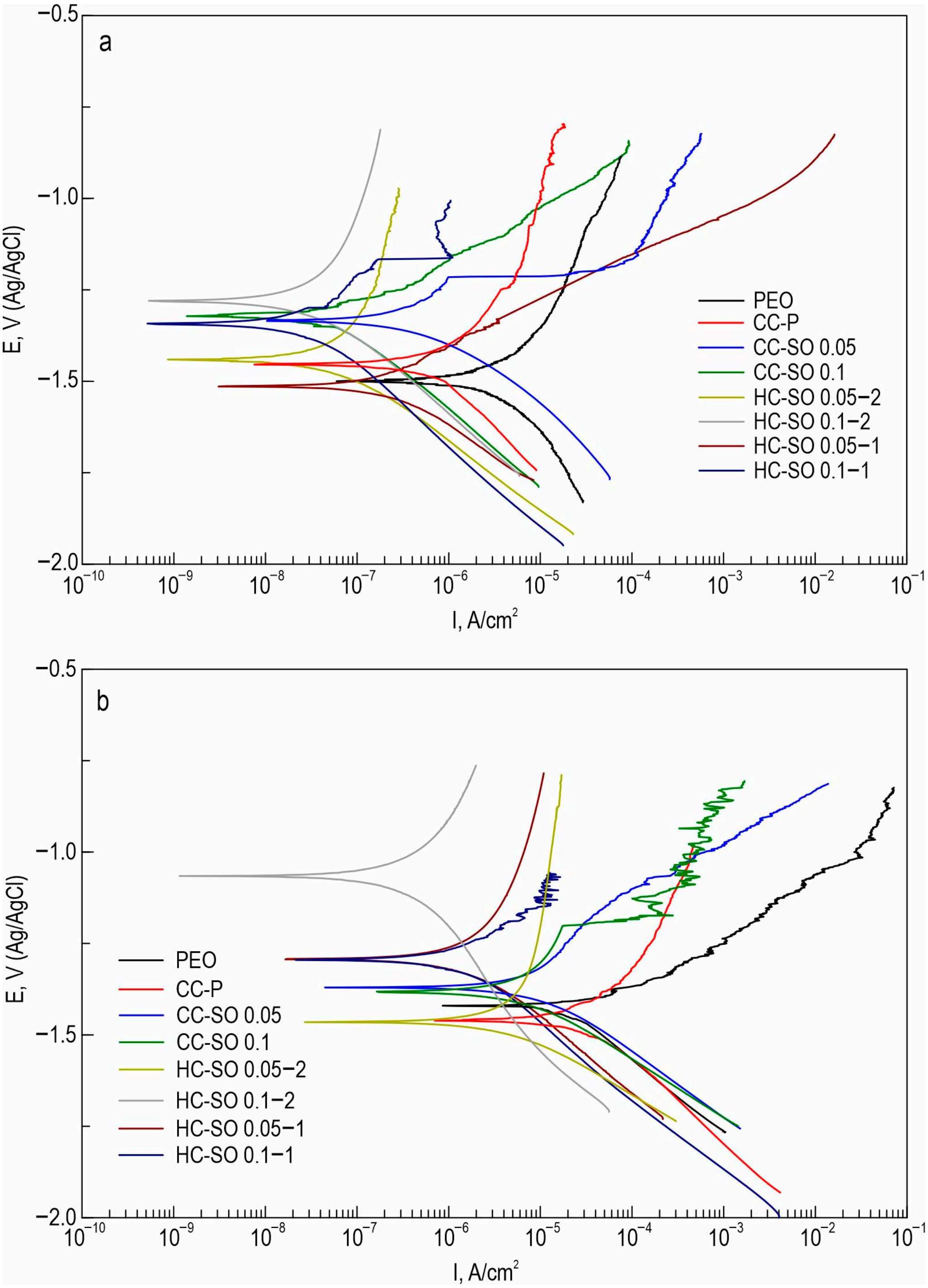

Figure S1. Polarization curves obtained after 1h (a) and 24 h (b) of exposure of the studied samples to 0.9%NaCl solution

Table S1. The calculated parameters of the EEC elements, obtained by fitting the experimental impedance spectra of the coated samples during their exposure to a 0.9% NaCl solution

**Exposure time, h**	***CPE*₁**		**R_1, Ω·cm²**	***CPE*₂**		**R_2, Ω·cm²**
	**Q_1, S·cm^{-2}·s^n**	**n_1**		**Q_2, S·cm^{-2}·s^n**	**n_2**	
**PEO**						
1	8.41×10^{-7}	0.61	2897	6.46×10^{-7}	0.91	77,559
3	6.75×10^{-7}	0.62	2362	1.31×10^{-6}	0.81	73,670
5	7.78×10^{-7}	0.61	2243	1.31×10^{-6}	0.82	86,070
7	4.14×10^{-7}	0.65	1554	1.53×10^{-6}	0.80	67,398
9	4.20×10^{-7}	0.65	1589	1.23×10^{-6}	0.84	82,627
11	5.78×10^{-7}	0.63	1670	1.21×10^{-6}	0.83	65,201
13	4.55×10^{-7}	0.64	1481	2.17×10^{-6}	0.73	49,428
15	7.32×10^{-7}	0.61	1628	1.12×10^{-6}	0.86	63,934
17	4.70×10^{-7}	0.64	1266	1.77×10^{-6}	0.78	50,641
19	9.11×10^{-7}	0.58	1554	1.09×10^{-6}	0.85	69,427
21	9.33×10^{-7}	0.57	1701	1.36×10^{-6}	0.86	78,171
23	4.53×10^{-7}	0.54	1869	2.23×10^{-6}	0.95	60,522
**CC-P**						
1	4.41×10^{-9}	0.84	5229	7.68×10^{-7}	0.78	112,710
3	4.31×10^{-9}	0.84	7507	8.78×10^{-7}	0.77	91,727
5	4.47×10^{-9}	0.84	7558	9.33×10^{-7}	0.78	73,998
7	5.09×10^{-9}	0.83	7652	1.00×10^{-6}	0.74	109,420
9	4.87×10^{-9}	0.83	8574	1.10×10^{-6}	0.71	122,800
11	5.25×10^{-9}	0.82	9178	9.34×10^{-7}	0.75	112,040
13	4.84×10^{-9}	0.83	9449	1.18×10^{-6}	0.69	120,070
15	5.68×10^{-9}	0.81	9791	1.00×10^{-6}	0.74	108,650
17	5.45×10^{-9}	0.82	9961	1.38×10^{-6}	0.67	115,060
19	6.14×10^{-9}	0.81	10,241	1.37×10^{-6}	0.65	138,260
21	5.51×10^{-9}	0.81	10,846	1.22×10^{-6}	0.68	133,120
23	6.37×10^{-9}	0.80	11,011	1.39×10^{-6}	0.63	160,000
**CC-SO 0.05**						
1	4.09×10^{-8}	0.85	104	7.53×10^{-7}	0.81	239,180
3	3.00×10^{-8}	0.87	95	1.15×10^{-6}	0.88	52,501
5	9.05×10^{-8}	0.78	122	1.51×10^{-6}	0.89	56,584
7	2.65×10^{-8}	0.88	124	1.72×10^{-6}	0.88	76,668
9	1.21×10^{-8}	0.95	128	1.68×10^{-6}	0.88	106,680
11	8.81×10^{-8}	0.80	161	1.56×10^{-6}	0.89	129,100
13	7.25×10^{-8}	0.82	164	1.54×10^{-6}	0.89	140,970

15	7.94×10^{-8}	0.81	171	1.54×10^{-6}	0.88	151,520
17	8.71×10^{-8}	0.80	180	1.52×10^{-6}	0.88	143,040
19	9.24×10^{-8}	0.80	180	1.57×10^{-6}	0.88	128,860
21	8.87×10^{-8}	0.82	175	1.56×10^{-6}	0.87	131,800
23	1.68×10^{-8}	0.92	154	1.68×10^{-6}	0.87	127,100
**CC-SO 0.1**						
1	3.41×10^{-7}	0.81	19223	2.53×10^{-7}	0.81	405,580
3	2.89×10^{-7}	0.95	7095	6.35×10^{-7}	0.76	143,710
5	8.84×10^{-7}	0.90	5766	1.62×10^{-6}	0.42	109,320
7	1.20×10^{-6}	0.90	6243	2.99×10^{-6}	0.25	98,219
9	1.23×10^{-6}	0.89	7889	1.33×10^{-6}	0.37	117,940
11	1.35×10^{-6}	0.89	8326	1.43×10^{-6}	0.35	137,430
13	1.32×10^{-6}	0.90	9554	1.66×10^{-6}	0.32	175,010
15	1.30×10^{-6}	0.90	11,669	1.66×10^{-6}	0.31	177,060
17	1.32×10^{-6}	0.89	11,833	2.08×10^{-6}	0.22	188,890
19	1.37×10^{-6}	0.89	7890	2.03×10^{-6}	0.22	188,050
21	1.36×10^{-6}	0.87	6769	1.72×10^{-6}	0.31	59,374
23	1.37×10^{-6}	0.89	11,514	5.05×10^{-6}	0.37	93,942
**HC-SO 0.05–2**						
1	2.20×10^{-9}	0.92	1436	4.21×10^{-7}	0.62	1011,000
3	3.07×10^{-9}	0.92	1443	5.38×10^{-7}	0.64	911,680
5	4.48×10^{-9}	0.90	1528	4.26×10^{-7}	0.71	774,530
7	4.52×10^{-9}	0.90	1345	5.17×10^{-7}	0.72	734,190
9	4.31×10^{-9}	0.90	1406	5.83×10^{-7}	0.72	701,280
11	4.23×10^{-9}	0.90	1442	6.04×10^{-7}	0.73	699,990
13	4.12×10^{-9}	0.91	1460	6.18×10^{-7}	0.73	651,290
15	4.00×10^{-9}	0.91	1480	6.30×10^{-7}	0.74	628,750
17	3.90×10^{-9}	0.91	1496	6.41×10^{-7}	0.74	608,110
19	1.25×10^{-9}	0.98	1435	6.27×10^{-7}	0.75	579,030
21	1.22×10^{-9}	0.99	1434	6.53×10^{-7}	0.75	566,180
23	1.19×10^{-9}	0.99	1476	6.69×10^{-7}	0.75	565,200
**HC-SO 0.1–2**						
1	2.71×10^{-9}	0.85	19,098	5.44×10^{-7}	0.65	247,030
3	2.88×10^{-9}	0.84	22,179	5.21×10^{-7}	0.65	402,780
5	3.36×10^{-9}	0.83	26,352	4.92×10^{-7}	0.66	500,280
7	2.27×10^{-9}	0.86	26,566	5.58×10^{-7}	0.62	646,960
9	2.16×10^{-9}	0.86	28,828	5.52×10^{-7}	0.62	848,100
11	2.40×10^{-9}	0.86	31,446	5.08×10^{-7}	0.65	1210,600
13	2.29×10^{-9}	0.86	32,837	5.49×10^{-7}	0.63	1676,700

15	1.62×10^{-9}	0.89	33,876	5.91×10^{-7}	0.60	1817,200
17	1.56×10^{-9}	0.89	32,006	6.14×10^{-7}	0.59	1776,700
19	3.15×10^{-9}	0.83	30,713	6.31×10^{-7}	0.59	1315,500
21	5.93×10^{-9}	0.79	32,975	6.08×10^{-7}	0.61	860,470
23	2.45×10^{-9}	0.85	29,210	5.93×10^{-7}	0.62	761,240
**HC-SO 0.05–1**						
1	1.56×10^{-9}	0.99	614	2.00×10^{-6}	0.57	56,211
3	1.50×10^{-9}	0.99	552	1.81×10^{-6}	0.57	58,201
5	4.07×10^{-9}	0.92	996	1.33×10^{-6}	0.61	80,697
7	3.48×10^{-9}	0.92	1204	1.24×10^{-6}	0.61	90,980
9	2.71×10^{-9}	0.93	1416	1.13×10^{-6}	0.62	111,980
11	2.44×10^{-9}	0.93	1706	1.11×10^{-6}	0.62	141,110
13	2.03×10^{-9}	0.94	2025	1.05×10^{-6}	0.62	145,430
15	2.11×10^{-9}	0.94	1978	1.05×10^{-6}	0.62	136,070
17	1.82×10^{-9}	0.94	2589	9.63×10^{-7}	0.62	280,970
19	1.71×10^{-9}	0.94	2777	1.01×10^{-6}	0.60	190,780
21	1.63×10^{-9}	0.95	2985	1.04×10^{-6}	0.60	170,060
23	1.63×10^{-9}	0.95	3211	1.05×10^{-6}	0.60	165,810

**HC-SO 0.1–1**									
**Exposure time, h**	***CPE*₁**		***R*₁, Ω·cm²**	***CPE*₂**		***R*₂, Ω·cm²**	***CPE*₃**		***R*₃, Ω·cm²**
	**Q_1, S·cm^{-2}·s^n**	**n_1**		**Q_2, S·cm^{-2}·s^n**	**n_2**		**Q_3, S·cm^{-2}·s^n**	**n_3**	
1	8.98×10^{-9}	0.88	312	4.49×10^{-7}	0.68	124,700	3.79×10^{-7}	0.70	2929
3	3.82×10^{-9}	0.91	480	4.37×10^{-7}	0.66	438,340	5.55×10^{-7}	0.65	7803
5	9.41×10^{-9}	0.88	1063	2.36×10^{-7}	0.82	338,560	7.00×10^{-7}	0.62	28,090
7	3.84×10^{-8}	0.76	1736	2.96×10^{-7}	0.83	362,960	5.27×10^{-7}	0.64	25,806
9	5.09×10^{-8}	0.73	2577	3.32×10^{-7}	0.84	371,390	4.80×10^{-7}	0.65	25,046
11	6.29×10^{-8}	0.72	3187	3.34×10^{-7}	0.85	406,640	4.79×10^{-7}	0.64	23,487
13	5.86×10^{-8}	0.72	3503	3.47×10^{-7}	0.85	401,680	4.66×10^{-7}	0.64	21,446
15	5.33×10^{-8}	0.73	3783	3.65×10^{-7}	0.86	405,280	4.73×10^{-7}	0.63	20,419
17	5.87×10^{-8}	0.72	4348	3.66×10^{-7}	0.86	406,900	4.83×10^{-7}	0.63	19,018
19	5.80×10^{-8}	0.72	4742	3.15×10^{-7}	0.87	672,940	4.83×10^{-7}	0.62	20,057
21	6.85×10^{-8}	0.70	6108	4.08×10^{-7}	0.83	681,510	3.14×10^{-7}	0.69	17,759
23	6.33×10^{-8}	0.71	6155	3.93×10^{-7}	0.83	692,750	3.63×10^{-7}	0.67	18,121

Table S2. The results of gravimetric measurements of specimens with different types of coatings after 7 days immersion in HBSS

**PEO**				
**Sample (#)**	**1**	**2**	**3**	**4**

Weight before exposure, g	0.5949	0.6908	0.5903	0.6393
Weight after exposure, g	0.5929	0.6887	0.5885	0.6370
Δ, g	0.0020	0.0021	0.0018	0.0023
Weight loss, $mg \cdot cm^{-2} \cdot d^{-1}$	0.0405	0.0426	0.0365	0.0466
**CC-P**				
**Sample (#)**	**1**	**2**	**3**	**4**
Weight before exposure, g	0.5491	0.6727	0.6919	0.6998
Weight after exposure, g	0.5502	0.6736	0.6929	0.7007
Weight increase, g	0.0011	0.0009	0.0010	0.0009
**CC-SO 0.05**				
**Sample (#)**	**1**	**2**	**3**	**4**
Weight before exposure, g	0.6292	0.6286	0.6467	0.6438
Weight after exposure, g	0.6234	0.6232	0.6404	0.6389
Δ. g	0.0058	0.0054	0.0063	0.0049
Weight loss, $mg \cdot cm^{-2} \cdot d^{-1}$	0.1175	0.1094	0.1277	0.0993
**CC-SO 0.1**				
**Sample (#)**	**1**	**2**	**3**	**4**
Weight before exposure, g	0.6616	0.6517	0.7033	0.6216
Weight after exposure, g	0.6573	0.6491	0.6983	0.6208
Δ, g	0.0043	0.0026	0.0050	0.0008
Weight loss, $mg \cdot cm^{-2} \cdot d^{-1}$	0.0871	0.0527	0.1013	0.0162
**HC-SO 0.05–2**				
**Sample (#)**	**1**	**2**	**3**	**4**
Weight before exposure, g	0.6112	0.6168	0.6113	0.6819
Weight after exposure, g	0.6089	0.6137	0.6084	0.6787
Δ, g	0.0023	0.0031	0.0029	0.0032
Weight loss, $mg \cdot cm^{-2} \cdot d^{-1}$	0.0466	0.0628	0.0588	0.0648
**HC-SO 0.1–2**				
**Sample (#)**	**1**	**2**	**3**	**4**
Weight before exposure, g	0.6734	0.6736	0.6007	0.6671
Weight after exposure, g	0.6728	0.6731	0.5999	0.6664
Δ, g	0.0006	0.0005	0.0008	0.0007
Weight loss, $mg \cdot cm^{-2} \cdot d^{-1}$	0.0122	0.0101	0.0162	0.0141
**HC-SO 0.05–1**				
**Sample (#)**	**1**	**2**	**3**	**4**
Weight before exposure, g	0.6820	0.6924	0.6991	0.7145
Weight after exposure, g	0.6805	0.6910	0.6972	0.7117
Δ, g	0.0015	0.0014	0.0019	0.0028

Weight loss, mg·cm^{-2}·d^{-1}	0.0304	0.0284	0.0385	0.0567
**HC-SO 0.1–1**				
**Sample (#)**	**1**	**2**	**3**	**4**
Weight before exposure, g	0.6783	0.6848	0.7317	0.7405
Weight after exposure, g	0.6743	0.6819	0.7268	0.7378
Δ, g	0.0040	0.0029	0.0049	0.0027
Weight loss, mg·cm^{-2}·d^{-1}	0.0810	0.0588	0.0993	0.0547

Table S3. The calculated parameters of the EEC elements, obtained by fitting the experimental impedance spectra of the coated AT-Mg samples during their exposure to 0.9 % NaCl solution for 24 h

**Exposure time, h**	***CPE$_1$***		**R_1, Ω·cm^2**	***CPE$_2$***		**R_2, Ω·cm^2**
	**Q_1, S·cm^{-2}·s^n**	**n_1**		**Q_2, S·cm^{-2}·s^n**	**n_2**	
**AT-Mg+PEO**						
1	2.50×10^{-6}	0.49	252	9.58×10^{-5}	0.47	795
3	1.20×10^{-6}	0.52	179	9.47×10^{-5}	0.69	290
5	1.08×10^{-6}	0.50	129	9.47×10^{-4}	0.82	141
**AT-Mg+CC SO**						
1	1.89×10^{-8}	0.87	124	2.13×10^{-5}	0.51	2226
3	6.10×10^{-9}	0.95	158	1.65×10^{-4}	0.53	412
5	3.67×10^{-9}	0.99	102	1.28×10^{-4}	0.79	129
**AT-Mg+HC SO**						
1	5.48×10^{-10}	0.93	8549	7.14×10^{-6}	0.21	63,813
3	5.14×10^{-10}	0.94	11,940	8.80×10^{-6}	0.33	30,995
5	4.34×10^{-10}	0.95	12,752	9.20×10^{-6}	0.29	40,040
7	4.71×10^{-10}	0.95	13,648	8.94×10^{-6}	0.34	34,488
9	4.16×10^{-10}	0.96	14,976	9.98×10^{-6}	0.34	32,817
11	4.30×10^{-10}	0.95	13,139	1.62×10^{-5}	0.29	24,732
13	4.25×10^{-10}	0.95	12,270	2.12×10^{-5}	0.29	23,097
15	4.04×10^{-10}	0.96	10,476	2.32×10^{-5}	0.28	19,948
17	5.10×10^{-10}	0.94	6339	5.22×10^{-5}	0.26	17,148
19	9.77×10^{-10}	0.89	4577	6.25×10^{-5}	0.31	9667
21	4.30×10^{-10}	0.97	3048	8.03×10^{-5}	0.31	3414
23	1.04×10^{-9}	0.91	1763	4.47×10^{-5}	0.57	1345
